# Management Patterns and Outcomes for Relapsed or Refractory Hodgkin Lymphoma

**DOI:** 10.14740/jh2218

**Published:** 2026-08-31

**Authors:** Arun G. Paniker, Joseph P. Marshalek, Eugene Chao, David Yashar, Sarah Tomassetti

**Affiliations:** a Department of Medicine, Harbor-UCLA Medical Center Torrance, CA 90502, USA; b Division of Hematology and Medical Oncology, Department of Medicine, Harbor-UCLA Medical Center Torrance, CA 90502, USA

**Keywords:** Hodgkin lymphoma, Relapsed/refractory, Brentuximab vedotin, Immune checkpoint inhibitor, Safety-net, Stem cell transplant, Salvage therapy, Real-world

## Abstract

**Background:**

Relapsed and refractory classical Hodgkin lymphoma (r/r cHL) remains a therapeutic challenge, particularly in underserved populations with limited access to novel agents. Data on management patterns and outcomes in safety-net health systems are sparse.

**Methods:**

We conducted a retrospective review of patients with r/r cHL treated at a Los Angeles County safety-net hospital between 2014 and 2024. Treatment regimens, response rates, and toxicities were analyzed.

**Results:**

Twenty-one patients with r/r cHL were included. Doxorubicin + bleomycin + vinblastine + dacarbazine was the predominant first-line regimen (85.7%). In the relapsed/refractory setting, outcomes differed by treatment class. Cytotoxic chemotherapy yielded an overall response rate (ORR) of 50% and complete response rate (CRR) of 25%. In comparison, immune checkpoint inhibitor (ICI) or brentuximab vedotin (BV) monotherapy demonstrated an ORR of 58% and CRR of 42%, while ICI/BV combination regimens achieved the highest observed response rates (ORR 100%, CRR 75%). One-year progression-free survival (PFS) was 38% with cytotoxic chemotherapy, 39% with ICI or BV monotherapy, and 63% with combination regimens. Median follow-up was 38 months, and overall survival (OS) for the entire cohort was 95% at 1 year and 85% at 5 years.

**Conclusions:**

In this real-world safety-net cohort, ICI- and BV-based regimens, particularly in combination, were associated with higher response rates compared with cytotoxic chemotherapy. These findings are consistent with prior studies and support the evolving role of targeted and immunotherapy-based approaches in r/r cHL, while highlighting the importance of access to novel therapies in underserved populations.

## Introduction

Classical Hodgkin lymphoma (cHL) is a form of lymphoma that primarily involves B cells. It has a bimodal age distribution, most commonly occurring in young adults aged 20–40, with a second peak after the age of 55 [1]. In 2024, the estimated incidence of Hodgkin lymphoma in the United States was 8,570 cases with approximately 910 deaths annually, while globally there were 82,409 reported cases and 22,701 deaths in 2022 [2, 3].

The primary malignant cell is the Hodgkin and Reed–Sternberg (HRS) cell, which is embedded within a highly immunoregulatory tumor microenvironment that promotes immune evasion [4]. Constitutive activation of intracellular signaling pathways supports HRS cell survival, and these cells typically express surface CD15 and CD30 antigens [4, 5]. Chromosome 9p24.1 alterations and microenvironmental signaling promote programmed death-ligand 1 (PD-L1) and programmed death-ligand 2 (PD-L2) over-expression, contributing to T-cell exhaustion and impaired anti-tumor immunity, while additional disruptions in antigen presentation and cytokine signaling further limit immune recognition [4, 6]. These biologic features have directly informed the development and increasing clinical integration of CD30-directed therapy as well as immune checkpoint blockade in the management of cHL.

The treatment of Hodgkin lymphoma represents one of the most successful advances in oncology over the past 40 years, with survival rates increasing from 74% in 1975–1977 to 89% in 2013–2019 [3]. Despite therapeutic progress, among patients with advanced-stage disease, approximately 10–15% experience primary refractory disease and an additional 30–40% ultimately relapse [7]. At the time of relapse or refractory disease, the goal of salvage therapy is to induce remission, preferably a complete response, as a bridge to autologous stem cell transplantation (ASCT), as patients who achieve a complete response followed by ASCT have improved progression-free survival (PFS) and overall survival (OS) [8, 9]. Historically, relapsed/refractory (r/r) cHL has been treated with combinations of cytotoxic chemotherapies as a bridge to stem cell transplantation (SCT). However, over the last decade, the introduction of brentuximab vedotin (BV) and immune checkpoint inhibitors (ICIs), including nivolumab and pembrolizumab, has transformed the treatment landscape.

Herein, we describe treatment patterns, efficacy, and toxicity in a retrospective cohort of patients with r/r cHL treated at an urban safety-net hospital.

## Materials and Methods

### Study design

A retrospective chart review was conducted for adult patients with relapsed or refractory Hodgkin lymphoma treated at Harbor-UCLA Medical Center from 2014 to 2024. Exclusion criteria included nodular lymphocyte predominant subtype, complete response to first-line therapy without subsequent relapse, no chemotherapy prior to first relapse, supportive care alone at first relapse, and incomplete data. Data were collected on demographics, disease characteristics, treatment, response, PFS, and OS. PFS was defined as time from therapy initiation to disease progression, relapse, or death from any cause. OS was defined as time from primary refractory disease or first relapse to death from any cause. Histopathologic subtype was abstracted from pathology reports. Salvage regimens were categorized as second-line therapy (2L) or third-line and later therapy (3L+) according to treatment sequence after first-line therapy. Duration of first remission was defined as the interval from completion of first-line therapy to first documented relapse and was reported in months, rounded to the nearest whole month; patients with primary refractory disease were considered not to have achieved a first remission.

### Staging and response assessment

Lymphoma staging was performed based on the Lugano staging classification [10]. Response to therapy was assessed via positron emission tomography (PET), single-photon emission computed tomography (SPECT), computed tomography (CT), or a combination of functional imaging (PET or SPECT) and CT. Imaging modality depended on the year of therapy, logistical factors, and provider selection. The Lugano response criteria were used to describe therapeutic response [10]. Overall response rate (ORR) includes patients with complete response or partial response.

### Adverse event assessment

As a retrospective study, there was no standardized protocol for documentation of adverse events. Adverse event data were collected from retrospective review of physician notes and laboratory data. The severity of adverse events was graded according to the Common Terminology Criteria for Adverse Events version 5 [11].

### Statistical analysis

A Z-test for two population proportions was used to compare ORR and complete response rate (CRR) between groups. Kaplan–Meier curves were utilized to depict PFS and OS with censoring at time of last follow-up. For PFS, a log rank test was performed to assess for differences between curves. A two-sided P-value of < 0.05 was chosen as the level of significance.

This study was conducted in compliance with the ethical standards of Harbor-UCLA Medical Center and with the Helsinki Declaration. Harbor-UCLA Medical Center Institutional Review Board (IRB) exemption status was obtained. Written informed consent was waived due to the retrospective nature of the study, and this decision was approved by the IRB.

## Results

### Patient cohort

**Figure 1:**
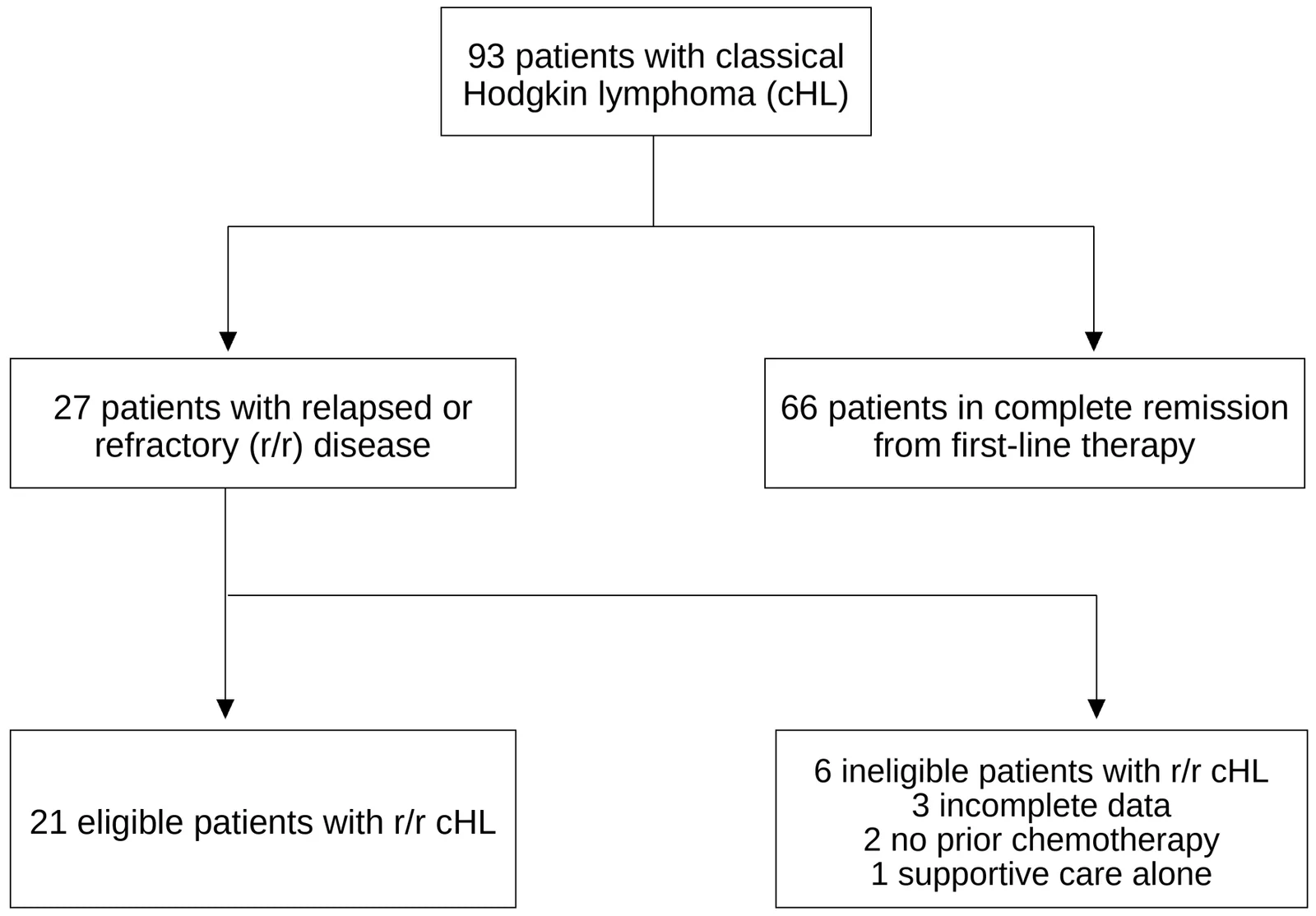
CONSORT diagram for patient inclusion. r/r: relapsed or refractory; cHL: classical Hodgkin lymphoma.

Among 93 patients with cHL, there were 27 patients with relapsed or refractory disease, and 21 who met inclusion criteria for the study (Fig. 1). Median age was 43 years (range 24–62). A majority of the patients were of Hispanic ethnicity (71%) or Black/African American (14%) (Table 1). Two (10%) patients were positive for human immunodeficiency virus. Most patients (18/21) received first-line doxorubicin + bleomycin + vinblastine + dacarbazine (ABVD). The cohort was composed of 14 patients with primary refractory disease and seven patients with relapsed Hodgkin lymphoma. Histopathologic subtypes included nodular sclerosis in eight patients (38%), mixed cellularity in five (24%), and unspecified cHL in eight (38%); no lymphocyte-rich or lymphocyte-depleted cases were identified. First-salvage ORRs were 50% for nodular sclerosis, 80% for mixed cellularity, and 75% for unspecified subtype. No clear relationship between histopathologic subtype and response was observed, and interpretation was limited by the small subgroup sizes and the proportion of cases without a specified subtype. Fourteen patients had primary refractory disease and therefore did not achieve a first remission. Among the seven patients with relapsed disease, the median duration of first remission was 61 months (range, 10–165 months).

**Table 1:** Demographics and Baseline Disease Characteristics

Characteristics	Number of patients (%)
Gender	
Male	10 (48)
Female	11 (52)
Age	
Median	43
Range	24–62
Race/ethnicity	
Hispanic	15 (71)
Black	3 (14)
White	1 (5)
Pacific Islander	1 (5)
Unknown	1 (5)
Stage	
I	1 (5)
II	9 (43)
III	2 (10)
IV	9 (43)
First-line treatment	
ABVD	18 (86)
BV-AVD	2 (10)
BEACOPP	1 (5)
Disease status	
Primary refractory	14 (67)
Relapsed	7 (33)
First remission duration (relapsed)^a^	
Median	61
Range	10–165
Histopathologic subtype	
Nodular sclerosis	8 (38)
Mixed cellularity	5 (24)
Lymphocyte-rich	0
Lymphocyte-depleted	0
Unspecified	8 (38)

^a^Individual durations were 16, 61, 12, 74, 10, 137, and 165 months. First remission duration was defined as the interval from completion of first-line therapy to first relapse. ABVD: doxorubicin + bleomycin + vinblastine + dacarbazine; BV-AVD: brentuximab vedotin + doxorubicin + vinblastine + dacarbazine; BEACOPP: bleomycin + etoposide + doxorubicin + cyclophosphamide + vincristine + procarbazine + prednisone.

### Treatment and efficacy

There were a total of 37 treatment regimens used across 21 patients, and the median number of lines of therapy for relapsed or refractory disease was 1 (range 1–5). The most common treatments were ifosfamide + carboplatin + etoposide (ICE) (n = 11), BV (n = 7), nivolumab + ICE (Nivo-ICE) (n = 4), and pembrolizumab (n = 4). Overall, 16 patients received a cytotoxic chemotherapy regimen without an ICI or BV, 12 patients received single-agent ICI or BV, and eight patients received combination therapy with an ICI or BV-based regimen (Table 2). Cytotoxic chemotherapy and ICI- or BV-based combination regimens were more commonly administered as second-line salvage therapy, whereas ICI or BV monotherapy was more commonly used in the third-line or later setting. Among cytotoxic chemotherapy regimens, 13/16 (81%) were administered as 2L and 3/16 (19%) as 3L+. Among ICI or BV monotherapy regimens, 3/12 (25%) were administered as 2L and 9/12 (75%) as 3L+. Among ICI- or BV-based combination regimens, 5/8 (63%) were administered as 2L and 3/8 (37%) as 3L+. Among regimens in these three treatment categories, 2L therapy consisted of 62% cytotoxic chemotherapy, 14% ICI or BV monotherapy, and 24% ICI- or BV-based combination regimens. The corresponding proportions for 3L+ therapy were 20%, 60%, and 20%, respectively. There were no significant differences in response rates or toxicity based on line of therapy.

**Table 2:** Therapies Used for Relapsed / Refractory Disease and Response Rates

	Complete response rate (CRR)	Overall response rate (ORR)
Cytotoxic chemotherapy (n = 16)	25%	50%
ICE (n = 11)	18%	55%
GemOx (n = 3)	33%	33%
IGEV (n = 1)	100%	100%
DHAP (n = 1)	0%	0%
ICI/BV alone (n = 12)	42%	58%
BV (n = 7)	43%	71%
Pembrolizumab (n = 4)	50%	50%
Nivolumab (n = 1)	0%	0%
ICI/BV combination (n = 8)	75%	100%
Nivo-ICE (n = 4)	75%	100%
BV-Nivolumab (n = 1)	100%	100%
BV-ICE (n = 1)	100%	100%
Pembrolizumab-GVD (n = 1)	100%	100%
Pembrolizumab + vorinostat (n = 1)	0%	100%
Other (n = 1)		
Everolimus (n = 1)	NE	NE

BV: brentuximab vedotin; DHAP: dexamethasone + high-dose cytarabine + cisplatin; GemOx: gemcitabine + oxaliplatin; GVD: gemcitabine + vinorelbine + liposomal doxorubicin; ICE: ifosfamide + carboplatin + etoposide; ICI: immune checkpoint inhibitor; IGEV: ifosfamide + gemcitabine + vinorelbine; Nivo-ICE: nivolumab + ifosfamide + carboplatin + etoposide; NE: not evaluable.

**Figure 2:**
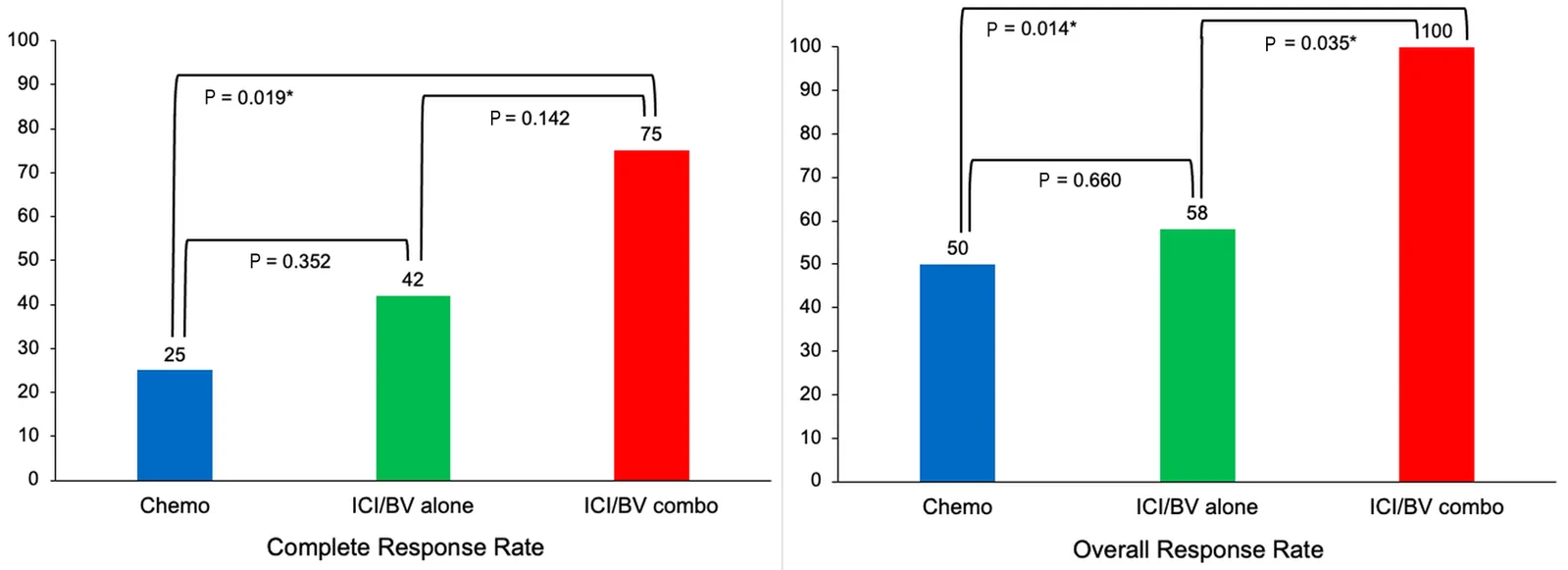
Complete response rate (CRR) and overall response rate (ORR) stratified by cytotoxic chemotherapy (Chemo), immune checkpoint inhibitor (ICI) or brentuximab vedotin (BV) alone (ICI/BV alone), and ICI/BV combination regimens (ICI/BV combo).

The CRR with cytotoxic chemotherapy was 25%, and the ORR was 50% (Fig. 2). Patients treated with ICI or BV monotherapy had a CRR of 42% and ORR of 58%. ICI/BV combination regimens had a CRR of 75%, which was significantly higher than cytotoxic chemotherapy (P = 0.019), and an ORR of 100%, which was significantly higher than cytotoxic chemotherapy (P = 0.014) and ICI or BV monotherapy (P = 0.035). There were no significant differences in CRR or ORR between cytotoxic chemotherapy and ICI or BV monotherapy (Fig. 2).

Nineteen (90%) patients were referred for evaluation for ASCT, and 14 (67%) patients received an ASCT. One patient also received an allogeneic SCT. Four patients received an additional eight lines of therapy at an outside facility which were not included in the efficacy analysis.

**Figure 3:**
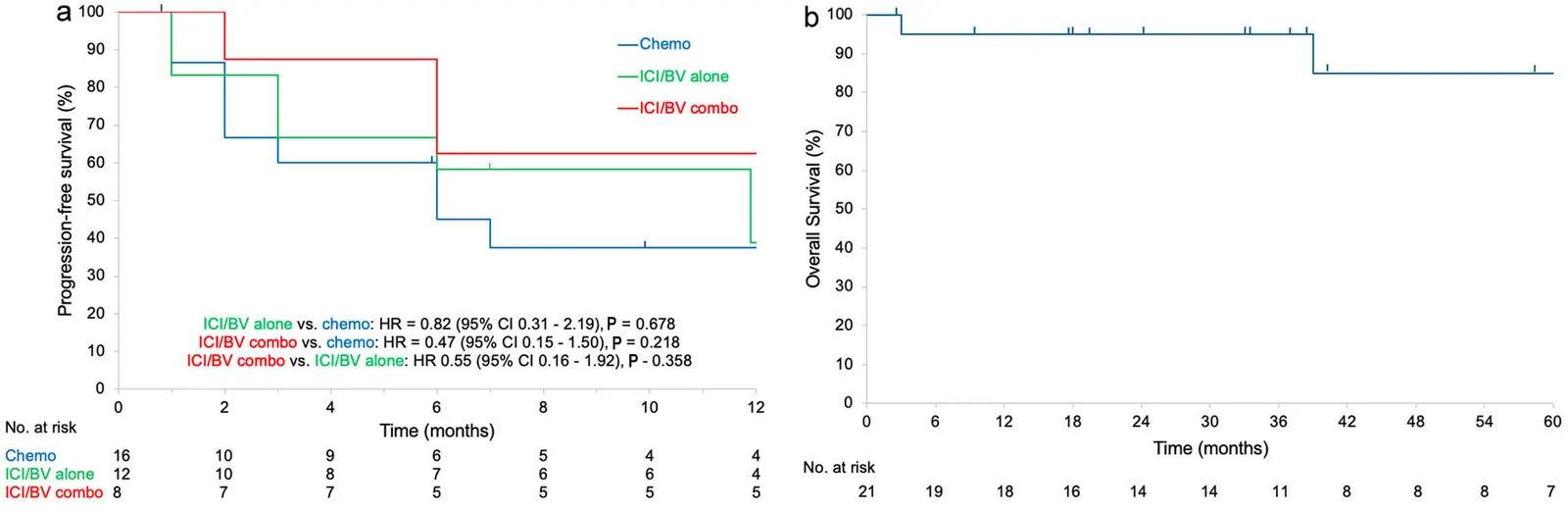
Kaplan-Meier curves for (a) progression-free survival (PFS) stratified by cytotoxic chemotherapy (Chemo), immune checkpoint inhibitor (ICI) or brentuximab vedotin (BV) alone (ICI/BV alone), and ICI/BV combination regimens (ICI/BV combo); (b) overall survival (OS) for all patients.

One-year PFS was 38% with cytotoxic chemotherapy, 39% with ICI or BV monotherapy, and 63% for ICI/BV combination regimens. There were no statistically significant differences in PFS between the three groups (Fig. 3a). The median duration of follow-up was 38 months. For the entire cohort, OS was 95% at 1 year and 85% at 5 years (Fig. 3b).

### Adverse events

Adverse events were observed in 35/37 (95%) treatment regimens, and grade 3 or worse adverse events were observed in 6/37 (16%) regimens. There were no significant differences in the risk of any grade or grade 3 or worse adverse events between ICE (82%, 36%), BV (100%, 29%), Nivo-ICE (100%, 25%), and pembrolizumab (100%, 0%). A complete list of adverse events observed with these four regimens can be found in Table 3. Cytopenias, transaminitis, and hematuria were observed with ICE and Nivo-ICE. The risk of peripheral sensory neuropathy was highest with BV (43%); however, no grade 3 or worse cases were observed. Pembrolizumab was associated with fatigue (50%) and dermatologic toxicity. The majority of grade 3 or worse adverse events were hematologic. One patient receiving Nivo-ICE died of multi-organ failure and hemophagocytic lymphohistiocytosis (HLH), which may have been treatment-related. There were no other potential treatment-related deaths. Adverse events resulting in treatment discontinuation were observed in one patient receiving ICE (grade 2 hematuria) and one patient receiving everolimus (grade 2 fatigue, grade 2 nausea).

**Table 3:** Adverse Events (AEs) Observed With the Four Most Common Regimens in Number (%) of Patients

	ICE (n = 11)		Brentuximab vedotin (n = 7)		Nivo-ICE (n = 4)		Pembrolizumab (n = 4)	
	Any grade	Grade 3 or worse	Any grade	Grade 3 or worse	Any grade	Grade 3 or worse	Any grade	Grade 3 or worse
Overall	9 (82)	4 (36)	7 (100)	2 (29)	4 (100)	1 (25)	4 (100)	0
Blood and lymphatic								
Febrile neutropenia	1 (9)	1 (9)	0	0	1 (25)	1 (25)	0	0
Neutropenia	4 (36)	4 (36)	1 (14)	0	2 (50)	1 (25)	0	0
Thrombocytopenia	5 (45)	2 (18)	1 (14)	0	3 (75)	1 (25)	0	0
Cardiac								
Atrial fibrillation	0	0	0	0	1 (25)	1 (25)	0	0
Heart failure	0	0	0	0	1 (25)	0	0	0
Endocrine								
Hypothyroidism	0	0	0	0	1 (25)	0	0	0
Gastrointestinal								
Constipation	2 (18)	0	0	0	0	0	0	0
Gastritis	0	0	0	0	0	0	1 (25)	0
Hyperbilirubinemia	0	0	2 (29)	0	1 (25)	0	0	0
Nausea	4 (36)	0	4 (57)	0	1 (25)	0	0	0
Transaminitis	3 (27)	0	1 (14)	0	2 (50)	1 (25)	0	0
Vomiting	1 (9)	0	1 (14)	0	0	0	0	0
General								
Fatigue	1 (9)	0	1 (14)	0	3 (75)	0	2 (50)	0
Infections								
Conjunctivitis	1 (9)	0	0	0	0	0	0	0
Kidney Infection	0	0	1 (14)	1 (14)	0	0	0	0
Musculoskeletal								
Back pain	0	0	0	0	0	0	1 (25)	0
Bone pain	0	0	0	0	0	0	1 (25)	0
Muscle cramp	1 (9)	0	0	0	0	0	0	0
Myalgias	1 (9)	0	0	0	1 (25)	0	0	0
Nervous system								
Dizziness	0	0	0	0	1 (25)	0	0	0
Dysgeusia	0	0	1 (14)	0	0	0	0	0
Encephalopathy	1 (9)	0	0	0	0	0	0	0
Headache	1 (9)	0	0	0	0	0	1 (25)	0
Peripheral sensory neuropathy	1 (9)	0	3 (43)	0	1 (25)	0	0	0
Renal								
Acute kidney injury	1 (9)	1 (9)	1 (14)	1 (14)	0	0	0	0
Hematuria	3 (27)	0	0	0	1 (25)	0	0	0
Skin								
Alopecia	1 (9)	0	0	0	0	0	0	0
Palmar plantar erythrodysesthesia	1 (9)	0	0	0	0	0	0	0
Pruritus	0	0	0	0	0	0	2 (50)	0
Rash acneiform	0	0	0	0	0	0	1 (25)	0

ICE: ifosfamide + carboplatin + etoposide.

## Discussion

### Overall treatment and efficacy

This study characterizes contemporary management strategies and treatment outcomes for patients with r/r cHL treated within a Los Angeles County safety-net health system. The analysis spans a period of substantial therapeutic transition marked by the introduction of targeted agents and immunotherapy. Historically, salvage management relied primarily on cytotoxic chemotherapy followed by ASCT; however, the incorporation of BV and anti–PD-1 therapies has expanded therapeutic options and altered treatment sequencing in relapsed disease [12–14]. Outcomes in this cohort were encouraging within the context of an underserved population and reflect evolving therapy availability.

### Treatment patterns and regimen categories

Therapy selection across the study period reflected transitions in both frontline and salvage approaches as newer agents were incorporated into clinical practice. ABVD remained the most common frontline regimen, with gradual incorporation of BV-AVD for advanced disease following publication of the ECHELON-1 trial [15]. In the relapsed/refractory setting, ICE was the most frequently utilized initial salvage regimen and predominated earlier in the study period. Over time, BV- and ICI-based approaches were incorporated into earlier lines of therapy, consistent with growing evidence supporting their use in earlier salvage settings and as bridges to transplantation [12, 13]. In the current cohort, cytotoxic chemotherapy and ICI/BV combination regimens were more frequently used as 2L salvage therapy, whereas single-agent ICI or BV was more frequently reserved for 3L+ treatment. Despite these differences in sequencing, response rates and toxicity did not differ significantly by line of therapy.

### Response outcomes by treatment class

Treatment responses differed across therapeutic categories. In this cohort, targeted monotherapy with BV or ICIs was associated with higher CRR and ORR than cytotoxic chemotherapy; however, these differences did not reach statistical significance. In contrast, ICI/BV combination therapy produced higher response rates; CRR was significantly higher than with cytotoxic chemotherapy, while ORR was significantly higher than with both cytotoxic chemotherapy and monotherapy. Although PFS did not differ significantly between treatment classes, ICI/BV combination regimens demonstrated numerically higher 1-year PFS. Overall, these findings align with published data in the relapsed and refractory setting reporting improved response rates with BV- and ICI-based approaches, particularly when administered in combination [12–24].

Given the small sample size and variability in treatment sequencing, comparisons between individual regimens should be interpreted cautiously. Within these constraints, Nivo-ICE was comparable to prior reports supporting ICI-based combinations as effective bridging strategies to transplantation [16]. Similarly, BV-containing regimens yielded outcomes in line with phase II trials and real-world observational series [17–21]. Finally, pembrolizumab monotherapy also achieved response rates consistent with previously reported results for single-agent ICIs in relapsed and refractory disease [22, 23]. SCT remained an integral component of management for patients achieving disease control. Although detailed transplant outcomes were limited due to referral to outside institutions, consolidation with ASCT was feasible following both chemotherapy-based and ICI/BV-based salvage approaches. Collectively, these results suggest higher response rates with ICI/BV combination therapy, consistent with prior studies and their growing role in the relapsed and refractory setting.

### Adverse events

Toxicity patterns observed in this cohort were consistent with established adverse event profiles of the evaluated regimens [25–28]. Cytotoxic chemotherapy, particularly ICE-containing regimens, was associated with the greatest burden of hematologic toxicity, including frequent cytopenias and most grade 3 or worse events. BV demonstrated a distinct toxicity profile characterized primarily by peripheral sensory neuropathy without severe hematologic toxicity. ICI therapy was generally well tolerated, with predominantly low-grade constitutional and dermatologic toxicities, and no grade 3 or worse events observed with pembrolizumab monotherapy. One death, possibly treatment-related, involving multi-organ failure and HLH occurred in a patient receiving Nivo-ICE, highlighting the potential for rare but serious immune-mediated complications with ICI regimens [29, 30]. Given favorable survival expectations in r/r cHL, differences in toxicity profiles may meaningfully influence regimen selection even in the absence of clear survival differences. Although no statistically significant differences in adverse event rates were observed in our data, distinct toxicity patterns highlight important clinical trade-offs among cytotoxic, antibody–drug conjugate, and immunotherapy-based strategies.

### Patient population

This study provides important insight into outcomes within an underserved safety-net population. As a county safety-net hospital, our patient population largely represents individuals from lower socioeconomic backgrounds, a factor consistently associated with worse outcomes in Hodgkin lymphoma [31]. Furthermore, the majority of patients in this cohort were Hispanic or Black, groups shown to experience worse outcomes in Hodgkin lymphoma, even after adjustment for socioeconomic status [31, 32]. The predominance of Hispanic patients in this cohort likely reflects the demographic composition of the hospital’s surrounding safety-net catchment population rather than a known racial or ethnic susceptibility to cHL [33]. The favorable outcomes observed in this cohort are notable given the disparities previously reported in these populations. High rates of transplant referral likely contributed, although fragmented care and transitions to external institutions limited complete follow-up. These findings suggest that delivery of novel therapies and access to transplant can support meaningful disease control in resource-constrained settings.

### Limitations

Several limitations should be considered when interpreting these results. The retrospective design and small sample size limit statistical power and may introduce selection and information biases. Assessment of potential relationships between histopathologic subtype and treatment response was limited by the small number of patients within each subtype and the absence of a specified subtype in 38% of cases. Treatment regimens were non-randomized and influenced by clinical factors, drug availability, and evolving treatment standards over time. Heterogeneity in salvage regimens and variability in follow-up, particularly surrounding transplant referral to outside institutions, limited comprehensive assessment of long-term outcomes and adverse events. Finally, as BV- and ICI-based regimens are increasingly incorporated into frontline therapy, the applicability of these findings—derived largely from patients treated with ABVD in the frontline setting—may become more limited over time.

### Conclusion

Despite these limitations, this study provides real-world insight into contemporary management of r/r cHL within a safety-net healthcare system. The findings highlight increasing adoption of targeted and immunotherapy-based regimens, encouraging clinical outcomes within an underserved population, and the importance of access to novel therapies and SCT. Future efforts should focus on multicenter safety-net collaborations to increase sample size, prospective evaluation of combination therapy strategies, and interventions aimed at reducing barriers to consistent follow-up and clinical trial participation. As therapies continue to evolve, ongoing evaluation of treatment outcomes in underserved populations will remain essential for ensuring equitable advances in Hodgkin lymphoma care.

## Data Availability

Upon request to the primary author, de-identified raw data can be made available.

## Abbreviations

ABVD: doxorubicin + bleomycin + vinblastine + dacarbazine
ASCT: autologous stem cell transplantation
BV: brentuximab vedotin
BV-AVD: brentuximab vedotin + doxorubicin + vinblastine + dacarbazine
cHL: classical Hodgkin lymphoma
CRR: complete response rate
CT: computed tomography
CTCAE: Common Terminology Criteria for Adverse Events
DHAP: dexamethasone + high-dose cytarabine + cisplatin
GemOx: gemcitabine + oxaliplatin
GVD: gemcitabine + vinorelbine + liposomal doxorubicin
HLH: hemophagocytic lymphohistiocytosis
HRS: Hodgkin and Reed–Sternberg
ICI: immune checkpoint inhibitor
ICE: ifosfamide + carboplatin + etoposide
IGEV: ifosfamide + gemcitabine + vinorelbine
IRB: Institutional Review Board
NE: not evaluable
Nivo-ICE: nivolumab + ifosfamide + carboplatin + etoposide
ORR: overall response rate
OS: overall survival
PD-L1: programmed death-ligand 1
PD-L2: programmed death-ligand 2
PET: positron emission tomography
PFS: progression-free survival
r/r cHL: relapsed or refractory classical Hodgkin lymphoma
SCT: stem cell transplantation
SPECT: single-photon emission computed tomography
